# 4-Octyl Itaconate and Dimethyl Fumarate Induce Secretion of the Anti-Inflammatory Protein Annexin A1 via NRF2

**DOI:** 10.4049/jimmunol.2200848

**Published:** 2023-08-14

**Authors:** Ciana Diskin, Emily A. Day, Órlaith C. Henry, Juliana E. Toller-Kawahisa, Luke A. J. O’Neill

**Affiliations:** School of Biochemistry and Immunology, Trinity Biomedical Sciences Institute, Trinity College, Dublin, Ireland

## Abstract

Annexin A1 is a key anti-inflammatory effector protein that is involved in the anti-inflammatory effects of glucocorticoids. 4-Octyl itaconate (4-OI), a derivative of the endogenous metabolite itaconate, which is abundantly produced by LPS-activated macrophages, has recently been identified as a potent anti-inflammatory agent. The anti-inflammatory effects of 4-OI share a significant overlap with those of dimethyl fumarate (DMF), a derivate of another Krebs cycle metabolite fumarate, which is already in use clinically for the treatment of inflammatory diseases. In this study we show that both 4-OI and DMF induce secretion of the 33-kDa form of annexin A1 from murine bone marrow–derived macrophages, an effect that is much more pronounced in LPS-stimulated cells. We also show that this 4-OI– and DMF-driven annexin A1 secretion is NRF2-dependent and that other means of activating NRF2 give rise to the same response. Lastly, we demonstrate that the cholesterol transporter ABCA1, which has previously been implicated in annexin A1 secretion, is required for this process in macrophages. Our findings contribute to the growing body of knowledge on the anti-inflammatory effects of the Krebs cycle metabolite derivatives 4-OI and DMF.

## Key Points

- 4-OI and DMF induce annexin A1 secretion in LPS-stimulated macrophages.
- The induction of annexin A1 secretion by 4-OI and DMF requires ABCA1 and NRF2.
- 4-OI induces annexin A1 secretion in vivo.

## Introduction

Annexin A1, formerly known as lipocortin-1, is a 37-kDa protein that belongs to the annexin superfamily of proteins. It is induced by glucocorticoids (1) and is widely considered to be an anti-inflammatory effector protein. One well-characterized anti-inflammatory function of annexin A1 is the suppression of eicosanoid synthesis through inhibition of cytoplasmic phospholipase A_2_ (2, 3). Annexin A1 has also been demonstrated to restrict the migration (4) and adhesion (5) of neutrophils, in addition to inducing their apoptosis (6). It inhibits mast cell degranulation and cytokine secretion and therefore is protective in models of mast cell–driven diseases (7–9). In macrophages, annexin A1 has been shown to limit NO synthesis and induce IL-10 secretion (10), in addition to enhancing phagocytosis of apoptotic neutrophils (11). More recently, it has been shown that annexin A1 can activate AMP-activated protein kinase and can thereby promote muscle regeneration (12) and protect against cerebral ischemia-reperfusion injury (13). Additionally, activation of AMP-activated protein kinase has been shown to be anti-inflammatory in many immune cell types, including macrophages (14–16), especially in the context of a resolution phenotype (17). Taken together, annexin A1 is a critical regulator of resolution of inflammation through several distinct cellular targets and has been shown to be protective across multiple disease models.

Although some of the functions of annexin A1, such as cytosolic phospholipase A_2_ inhibition, are dependent on cytosolic annexin A1, it is also well documented that annexin A1 can be secreted (18). Once secreted, annexin A1 can bind to a family of G protein–coupled receptors named formyl peptide receptors (FPRs), particularly FPR2. Although annexin A1 has been reported to be secreted by a number of different cell types, it does not contain a canonical signal sequence for secretion (19). However, the ATP-binding cassette transporter A1 (ABCA1), the canonical function of which is cholesterol efflux (20), has been implicated in annexin A1 secretion. ABC transporters were first linked to annexin A1 secretion when it was reported that several ABC transporter inhibitors suppressed annexin A1 secretion in inflamed rat mucosa (21). It was also demonstrated using both pharmacological and genetic methods to target ABCA1 that annexin A1 secretion by pituitary folliculostellate cells was ABCA1-dependent (22). More recently, it was shown that secretion of annexin A1 via ABCA1 ameliorated retinal inflammation, with this study also demonstrating direct interaction between annexin A1 and ABCA1 (23). Although ABCA1-dependent annexin A1 secretion has not been explicitly shown for macrophages, they are known to express high levels of this transporter (24). Additionally, there have been reports of intracellular cleavage of annexin A1 into a 33-kDa fragment in neutrophils, which can be secreted (25, 26). However, a great deal remains to be clarified in relation to the regulation of annexin A1 secretion.

Macrophages are known to undergo extensive metabolic reprogramming upon stimulation with LPS. Two of the metabolic changes that occur under these conditions are the production of high concentrations of itaconate from the Krebs cycle intermediate *cis*-aconitate (27), and high concentrations of fumarate due to increases in aspartate-argininosuccinate shunt and reductions in fumarate hydratase (28). Itaconate is produced from *cis*-aconitate through a reaction that is catalyzed by the enzyme aconitate decarboxylase 1 (often called IRG1), which is potently upregulated by LPS (29, 30). The antibacterial effects of itaconate have been recognized for some time (30–32), but more recently itaconate has emerged as a key immunomodulator (33). 4-Octyl itaconate (4-OI) and dimethyl fumarate (DMF) are cell-permeable derivatives of itaconate and fumarate, respectively, and have been developed for studying the functions of these metabolites. Itaconate and 4-OI have been demonstrated to modify cysteine residues of proteins in a process termed 2,3-dicarboxypropylation, or itaconation (27). However, 4-OI is much more potent at targeting cysteine residues and has several distinct cellular targets from itaconate (34). Similarly, fumarate and DMF are known to modify cysteine residues in a process termed succination (not to be confused with succinylation) (35). Many anti-inflammatory functions of itaconate, 4-OI, fumarate, and DMF have been reported during the last number of years. It has been demonstrated that crucial cysteines of KEAP1 undergo this modification by 4-OI and DMF, which subsequently targets KEAP1 for proteasomal degradation (27, 36). This liberates the master antioxidant transcription factor NRF2, permitting induction of antioxidant genes and downregulation of proinflammatory cytokines such as pro–IL-1β (37). Itaconate and 4-OI have also been shown to limit NLRP3 inflammasome activation and pyroptosis (38, 39), impair glycolysis (40, 41), and inhibit TET DNA dioxygenases to suppress transcription of proinflammatory genes (42). Additionally 4-OI and DMF both target GAPDH (40, 43) and gasdermin D (39, 44, 45). Importantly, DMF is currently used clinically (trade name Tecfidera) for the treatment of multiple sclerosis (MS) and psoriasis (46) and is the most commonly prescribed medication for relapsing-remitting MS. Given the shared cysteine reactivity of 4-OI and DMF, using these compounds can help us better understand the role of cysteine modification in inflammatory/resolution processes, and potentially highlight uses of the cysteine-reactive compounds for the treatment of other inflammatory diseases.

The effect of 4-OI and DMF on annexin A1 or its secretion has not been studied to date. In this study, we demonstrate that both of these Krebs cycle metabolite derivatives induce secretion of the 33-kDa fragment of annexin A1 from murine bone marrow–derived macrophages (BMDMs). We provide evidence to indicate that the endogenous metabolites, itaconate and fumarate, do not recapitulate the effect of the derivatives, perhaps due to lower electrophilicity. We also show that the capacity of 4-OI and DMF to induce annexin A1 is dependent on NRF2 activation. Finally, we demonstrate that the transporter ABCA1 plays a role in 4-OI– and DMF-induced annexin A1 secretion. Our study therefore describes a phenomenon that could potentially contribute to the general anti-inflammatory profile of these compounds.

## Materials and Methods

### Reagents

LPS derived from *Escherichia coli,* serotype EH100 (Enzo Life Sciences, ALX-581-010), R848 (Sigma-Aldrich, SML0196), poly(I:C) (InvivoGen, tlrl-ic), Pam3CSK4 (InvivoGen, tlrl-pms), DMF (Sigma-Aldrich, 24226), diethyl maleate (DEM; Sigma-Aldrich, D97703), and fumarate hydratase-IN-1 (MedChemExpress, HY-100004) were used. 4-OI was initially supplied by Prof. Richard Hartley, but results were confirmed and later experiments conducted using commercially available 4-OI (Sigma-Aldrich, SML2338). Abs used were anti-annexin A1 (Cell Signaling Technology, 3299), anti-NRF2 (Cell Signaling Technology, 12721S), anti-KEAP1 (Cell Signaling Technology, 8047S), anti-ABCA1 (Cell Signaling Technology, 96292), anti–IL-1β (R&D Systems, AF401-NA), and anti–β-actin (Sigma-Aldrich, AC-74). Anti-mouse IgG and anti-rabbit IgG secondary HRP-conjugated Abs (Jackson ImmunoResearch) were also used. The Silencer Select small interfering RNAs (siRNAs) against NRF2 (s70522), KEAP1 (s78526), ABCA1 (s61785), and FH (s66045) in addition to the Silencer Select negative control (Thermo Fisher Scientific) were used.

### Mice and BMDM generation

BMDMs were isolated from C57BL/6OlaHSD mice (Harlan UK). Legs from NRF2 knockout mice and their matched wild types were provided by Prof. Albeena Dinakova-Kostova (University of Dundee). All animals were housed under specific pathogen-free conditions in accordance with Irish and European Union regulations. All experiments carried out were subject to prior ethical approval by the Trinity College Dublin Animal Research Ethics Committee and the Health Products Regulatory Authority. The mice were euthanized in a carbon dioxide chamber, followed by cervical dislocation as confirmation of death. The tibia, femur, and hip bones were cut at each end and the bone marrow was subsequently flushed with cell culture media. The cells were then differentiated in DMEM containing 10% (v/v) FCS, 1% (v/v) penicillin/streptomycin, and 20% (v/v) L929 supernatant for 6 d. After this time, the macrophages were counted and replated for use in experiments.

For in vivo experiments 10-wk-old female C57BL/6OlaHSD mice were i.p. injected with vehicle (20% β-cyclodextrin, 10% DMSO in PBS), 4-OI (50 mg/kg), or DMF (25 mg/kg). Two hours later mice were injected with PBS or LPS (2.5 mg/kg) and sacrificed 2 h later. Blood was collected by retro-orbital bleed following CO_2_ asphyxiation. Blood was allowed to clot at room temperature for 30 min and spun at 5000 relative centrifugal force for 10 min at 4°C. Serum was collected and stored and −80°C for future analysis. Annexin A1 was detected in the serum by ELISA (Assay Genie, MOEB0294).

### siRNA transfection

The media on the macrophages was replaced with DMEM that had not been supplemented with FCS, penicillin/streptomycin, or L929 supernatant. The required amounts of Lipofectamine RNAiMAX transfection reagent (Thermo Fisher Scientific) and the appropriate siRNA were both diluted in DMEM, mixed together, and then preincubated for 15 min. This mixture was then added to each well such that the final dilution of Lipofectamine RNAiMAX transfection reagent was 5 μl/ml and the final concentration of siRNA was 50 nM. Transfection was left for 8 h, when fresh medium was added (10% FCS, 1% penicillin/streptomycin, and 10% L929). For NRF2, siRNA cells were left overnight, and for KEAP1 and FH, siRNA cells were left for 48 h before the indicated treatments.

### Western blotting

For lysate blots, cells were lysed in sample buffer (0.125 M Tris [pH 6.8], 10% [v/v] glycerol, 0.02% SDS, 5% DTT) and thereafter incubated at 95°C for 5 min. For supernatant blots, 1 μl of StrataClean resin (Agilent Technologies) was added for every 100 μl of supernatant followed by a shaking incubation step at 4°C for 5 min. The supernatants were subsequently centrifuged at 210 × *g* for 2 min. The supernatants were discarded and 20 μl of sample lysis buffer was added to the pellet. The supernatant proteins were then eluted from the StrataClean resin by adding sample buffer and incubating the samples at 95°C for 5 min, followed by centrifugation at 210 × *g* for 2 min. The samples were resolved on SDS-polyacrylamide gels. On each gel the Spectra BR protein ladder (Thermo Fisher Scientific) was also run so that proteins could be identified according to molecular mass. The protein content of the gel was then transferred to polyvinylidene fluoride membranes, and membranes were subsequently blocked for 1 h in 5% (w/v) dried milk in TBST. The blots were then incubated with the primary Ab overnight at 4°C. Following a 1-h incubation with the secondary Ab at room temperature, as well as three washes in TBST before and after the secondary Ab was added, the blots were developed using chemiluminescent substrate (Thermo Fisher Scientific).

### Real-time PCR

Cells were lysed and the RNA was extracted using the PureLink RNA mini kit (Ambion). cDNA was prepared from the RNA samples using a high-capacity cDNA reverse transcription kit (Applied Biosystems), in accordance with the manufacturer’s instructions. Real-time quantitative PCR (qPCR) was then performed with the resulting cDNA using a 7500 fast real-time PCR system with PowerUp SYBR Green master mix (Applied Biosystems). All genes were normalized to *Rps18* expression. The sequences of the primer pairs for murine genes that were used are as follows: *Rsp18*, 5′-GGA TGT GAA GGA TGG GAA GT-3′ (forward) and 5′-CCC TCT ATG GGC TCG AAT TT-3′ (reverse); *Abca1*, 5′-CAGCACCGTGTCTTGTCTGA-3′ (forward) and 5′-GAGACATCGATGGTCAGCGT-3′ (reverse); *Anxa1*, 5′-ACA ACC ATC GTG AAG TGT GCA CAA CCA TCG TGA AGT GTG -3′ (forward) and 5′-ATT TCC GAA CGG GAG ACC AT-3′ (reverse); *Il1b*, 5′-GAG GAC ATG AGC ACC TTC TTT-3′ (forward), 5′-GCC TGT AGT GCA GTT GTC TAA-3′ (reverse); *Hmox1*, 5′-CCT CAAC AGA TGG CGT CAC TT-3′ (forward), 5′-GCT GAT CTG GGG TTT CCC TC-3′ (reverse).

### Statistical analysis

Statistical significance was determined by the one-way or two-way ANOVA methods as described in the figure legends. For the one-way ANOVA, data were analyzed with no matching or pairing. Gaussian distribution and equal SD were assumed. Multiple comparisons were performed comparing the mean of each column to the mean of every other column. The Tukey test was used for correction for multiple comparisons. For the two-way ANOVA, multiple comparisons were performed comparing each cell mean with the other cell mean in that row. The Šidák test was used for correction for multiple comparisons. Data are expressed as mean ± SEM. Significance was defined as follows: *p < 0.05, **p < 0.01, ***p < 0.001, ****p < 0.0001. GraphPad Prism v9 software was used for statistical analysis.

## Results

### 4-OI induces annexin A1 secretion by macrophages

**FIGURE 1.**
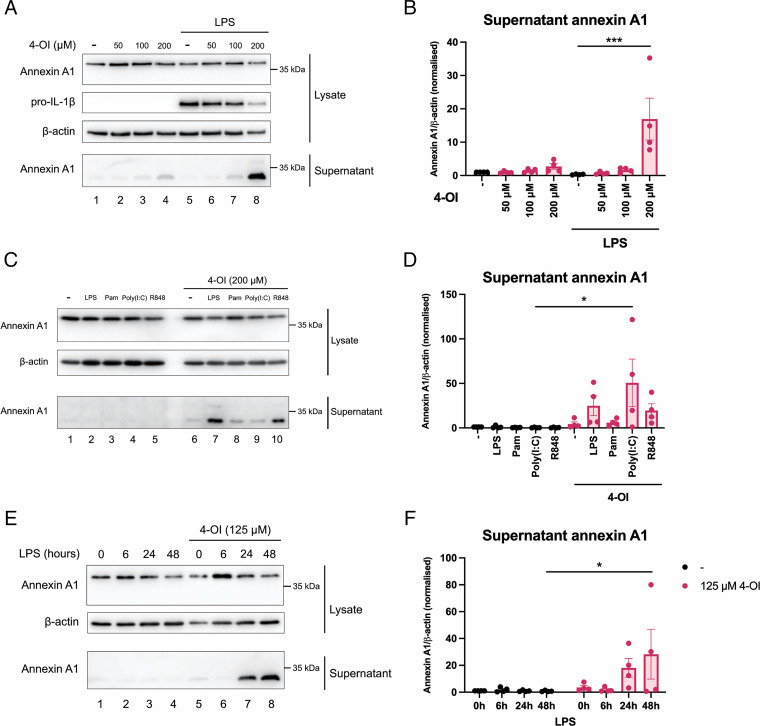
4-OI induces annexin A1 secretion from BMDMs. (**A**) Representative Western blots of BMDMs that were pretreated with 4-OI (50–200 μM) or vehicle for 2 h prior to stimulation with LPS (100 ng/ml) for 24 h. Cell lysates were harvested and cell supernatants were concentrated. (**B**) Quantification of supernatant annexin A1 (*n* = 4 from three independent experiments). (**C**) Representative Western blots of BMDMs that were pretreated with 200 μM 4-OI for 2 h prior to stimulation with LPS (100 ng/ml), Pam3CSK4 (100 ng/ml), poly(I:C) (1 μg/ml), or R848 (500 ng/ml) for 24 h. Cell lysates were harvested and cell supernatants were concentrated. (**D**) Quantification of supernatant annexin A1 (*n* = 4 from three independent experiments). (**E**) BMDMs were pretreated with 125 μM 4-OI for 2 h prior to stimulation with LPS (100 ng/ml) for 6, 24, or 48 h. (**F**) Supernatant annexin A1 levels were measured by Western blotting (*n* = 4 from three independent experiments). Cell lysates were harvested and cell supernatants were concentrated. Data are presented as mean ± SEM and a one-way or two-way ANOVA was performed. The data show the adjusted *p* value obtained from multiple comparisons, corrected for using the Tukey test for one-way ANOVA or Šidák test for two-way ANOVA. *p < 0.05, ***p < 0.0005.

As many anti-inflammatory effects have already been reported for 4-OI (33), and annexin A1 has long been recognized as a crucial anti-inflammatory effector, we investigated the effect of 4-OI on annexin A1 expression in LPS-stimulated BMDMs. Intracellular levels of annexin A1 were relatively unaffected (Fig. 1A, quantified in Supplemental Fig. 1A), but pretreatment with 4-OI gave rise to the presence of annexin A1 in the cell supernatant (Fig. 1A, top and bottom panels, lane 4 compared with lane 1, quantified in Fig. 1B). Although 4-OI does induce a low level of annexin A1 secretion in unstimulated cells, this effect is far more pronounced in the macrophages that had been stimulated with LPS supernatant (Fig. 1A, bottom panel, lane 8 compared with lane 5, quantified in Fig. 1B). As a control, we confirmed that 4-OI reduces pro–IL-1β expression (Fig. 1A, second panel, lane 8 compared with lane 5, quantified in Supplemental Fig. 1B), which we and others have previously demonstrated (27, 47). Notably, the annexin A1 detected in the supernatant is around 33 kDa, whereas in the lysate the band is present at the expected size of 37 kDa. There are reports in the literature of a cleaved form of annexin A1 measuring 33 kDa being secreted (25, 26). The effect of 4-OI on annexin A1 secretion was also more pronounced in cells stimulated with a wider range of TLR ligands (Fig. 1C, bottom panel, lanes 7–10 compared with lanes 2–5, quantified in Fig. 1D), and similarly, there was no effect on lysate annexin A1 (Supplemental Fig. 1C). We then tested several time points after LPS stimulation and found that 4-OI induced annexin A1 secretion at longer time points of 24 and 48 h but not as early as 6 h (Fig. 1E, bottom panel, lanes 7 and 8 compared with lane 6, quantified in Fig. 1F), again with no effects on lysate annexin A1 (Supplemental Fig. 1D). We next investigated whether endogenous itaconate would also impact annexin A1 secretion using BMDMs lacking *Irg1*, the gene encoding the enzyme for itaconate synthesis. However, we observed no difference when comparing supernatant annexin A1 levels between *Irg1^+/+^* and *Irg1*^−/−^ BMDMs (Supplemental Fig. 1E, bottom panel, lanes 1 and 2 compared with lanes 3 and 4, quantified in Supplemental Fig. 1F–H). From these data we can conclude that 4-OI induces annexin A1 secretion from murine macrophages, whereas endogenous itaconate does not. This observation was unsurprising because LPS alone, which would increase endogenous itaconate, does not induce annexin A1 secretion.

### DMF induces annexin A1 secretion by macrophages

**FIGURE 2.**
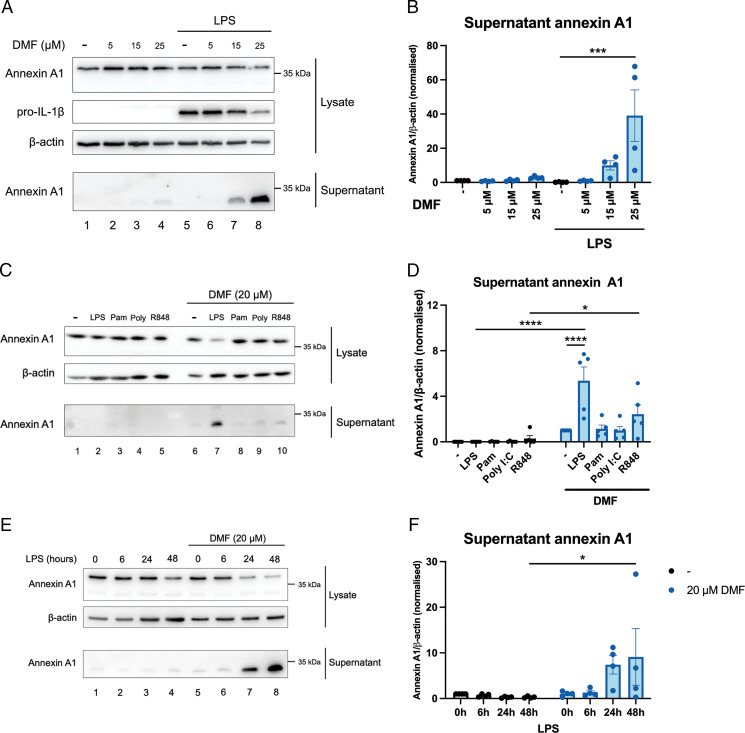
DMF induces annexin A1 secretion from BMDMs. (**A**) Representative Western blots from BMDMs pretreated with DMF (5–25 μM) or vehicle for 2 h prior to stimulation with LPS (100 ng/ml) for 24 h. Cell lysates were harvested and cell supernatants were concentrated. (**B**) Supernatant annexin A1 quantification (*n* = 4 from three independent experiments). (**C**) Representative Western blots from BMDMs pretreated with 20 μM DMF for 2 h prior to stimulation with LPS (100 ng/ml), Pam3CSK4 (100 ng/ml), poly(I:C) (1 μg/ml), or R848 (500 ng/ml) for 24 h. Cell lysates were harvested and cell supernatants were concentrated. (**D**) Quantification of supernatant annexin A1 (*n* = 5 from three independent experiments). (**E**) BMDMs were pretreated with 20 μM DMF for 2 h prior to stimulation with LPS (100 ng/ml) for 6, 24, or 48 h. (**F**) Supernatant annexin A1 levels were measured by Western blotting (*n* = 4 from three independent experiments). Data are presented as mean ± SEM, and a one-way or two-way ANOVA was performed. The data show the adjusted *p* value obtained from multiple comparisons, corrected for using the Tukey test for one-way ANOVA or Šidák test for two-way ANOVA. *p < 0.05, ***p < 0.0005, ****p < 0.0001.

We next tested whether DMF might have a similar effect on annexin A1 secretion to 4-OI. DMF, in a manner akin to 4-OI, induced secretion of the 33-kDa form of annexin A1, especially from LPS-stimulated macrophages (Fig. 2A, lane 8 compared with lane 5, quantified in Fig. 2B, Supplemental Fig. 2A). Again, as a control we show that DMF attenuates pro–IL-1β expression (Fig. 2A, lane 8 compared with lane 5, quantified in Supplemental Fig. 2B), which has previously been reported in the literature (43). Similar to 4-OI, DMF most strongly induced annexin A1 secretion when used in combination with LPS stimulation, although there were also increases seen when used in combination with R848 (Fig. 2C, bottom panel, lanes 7 and 10 compared with lane 6, quantified in Fig. 2D, Supplemental Fig. 2C). Similar to our results using 4-OI, DMF induced annexin A1 secretion at 24 and 48 h but not after only 6 h of stimulation with LPS (Fig. 2E, bottom panel, lanes 7 and 8 compared with lane 6, quantified in Fig. 2F, Supplemental Fig. 2D). We next tested whether endogenous fumarate would affect annexin A1 secretion. For this we used an inhibitor of the enzyme fumarate hydratase (FHin) to boost intracellular fumarate levels. Treatment with FHin did not significantly induce annexin A1 secretion (Supplemental Fig. 2E, bottom panel, lanes 5 and 6 compared with lane 4, quantified in Supplemental Fig. 2F, 2G). To further test the role of endogenous fumarate accumulation, we used genetic knockdown of fumarate hydratase to increase intracellular fumarate levels. Knockdown efficiency was ∼50% (Supplemental Fig. 2H, top panel, quantified in Supplemental Fig. 2I). We did not find any changes to secretion of annexin A1 with LPS treatment (Supplemental Fig. 2H, bottom panel, quantified in Supplemental Fig. 2J, 2K), suggesting that the effect we observed on annexin A1 secretion occurs for DMF but not endogenous fumarate.

### 4-OI and DMF induce annexin A1 secretion in an NRF2-dependent manner

**FIGURE 3.**
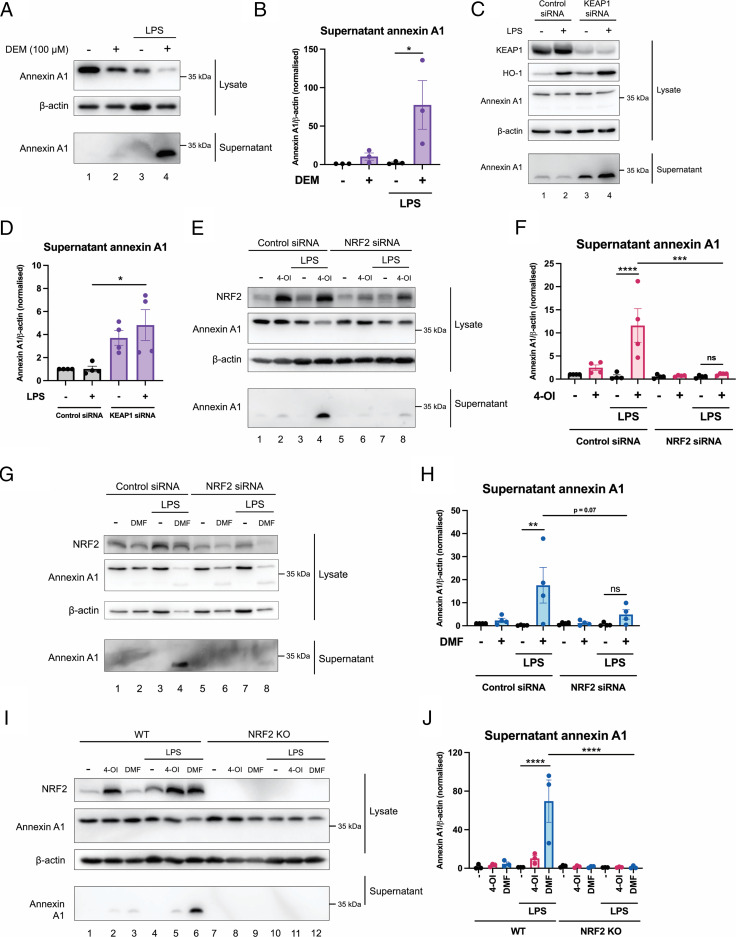
4-OI and DMF induce annexin A1 secretion in an NRF2-dependent manner. (**A**) BMDMs were pretreated with 100 μM DEM for 2 h prior to stimulation with LPS (100 ng/ml) for 24 h. Cell lysates were harvested and cell supernatants were concentrated. (**B**) Supernatant annexin A1 levels were measured by Western blotting (*n* = 3 from three independent experiments). (**C**) Representative Western blots from BMDMs that were transfected with 50 nM control siRNA or KEAP1 siRNA for 48 h, prior to stimulation with LPS (100 ng/ml) for 24 h. (**D**) Supernatant annexin A1 (*n* = 4 from three independent experiments). (**E**) Representative Western blots from BMDMs that were transfected with 50 nM control siRNA or NRF2 siRNA for 24 h. The cells were then pretreated with 200 μM 4-OI for 2 h prior to stimulation with LPS (100 ng/ml) for 24 h. (**F**) Quantification of supernatant annexin A1 (*n* = 4 from three independent experiments). (**G**) BMDMs were transfected with 50 nM control siRNA or NRF2 siRNA for 24 h. The cells were then pretreated with 25 μM DMF for 2 h prior to stimulation with LPS (100 ng/ml) for 24 h. (**H**) Quantification of supernatant annexin A1 (*n* = 4 from three independent experiments). (**I**) BMDMs from wild-type and NRF2 knockout mice were pretreated with vehicle, 200 μM 4-OI, or 25 μM DMF for 2 h prior to stimulation with LPS (100 ng/ml) for 24 h. Cell lysates were harvested and cell supernatants were concentrated. (**J**) Supernatant annexin A1 levels were measured by Western blotting (*n* = 3 from one independent experiment). Data are presented as mean ± SEM, and a one-way ANOVA was performed. The data show the adjusted *p* value obtained from multiple comparisons, corrected for using the Tukey test. *p < 0.05, **p < 0.005, ***p < 0.0005, ****p < 0.0001; ns, not significant.

As 4-OI and DMF have both been identified as potent NRF2 activators (27, 36), we sought to investigate whether the capacity of 4-OI and DMF to induce annexin A1 secretion was NRF2-dependent. We observed that an additional NRF2-activating compound, DEM, also promoted secretion of annexin A1 in LPS-stimulated BMDMs while reducing lysate annexin A1 in resting macrophages (Fig. 3A, bottom panel, lane 4 compared with lane 3, quantified in Fig. 3B, Supplemental Fig. 3A). We next took a genetic approach by knocking down the NRF2 regulator KEAP1, as 4-OI, DMF, and DEM all act via KEAP1 modification (27, 36, 48). Treatment of BMDMs with an siRNA against KEAP1 resulted in reduction of KEAP1 protein levels (Fig. 3C, top panel, lanes 3 and 4 compared with lanes 1 and 2, quantified in Fig. 3D, Supplemental Fig. 3B) and an increase in heme oxygenase-1 (HO-1), which is NRF2-dependent (Fig. 3C, second panel, lanes 3 and 4 compared with lanes 1 and 2, quantified in Supplemental Fig. 3C). KEAP1 knockdown also resulted in increased secretion of 33-kDa annexin A1, whereas lysate annexin A1 remained unchanged (Fig. 3C, bottom panel, lanes 3 and 4 compared with lanes 1 and 2, quantified in Fig. 3D, Supplemental Fig. 3D). To evaluate whether 4-OI and DMF could still induce annexin A1 secretion in the absence of NRF2, we knocked down NRF2 using siRNA. Silencing of NRF2 significantly impaired the ability of 4-OI (Fig. 3E, bottom panel, lane 8 compared with lane 4, quantified in Fig. 3F) and also DMF (Fig. 3G bottom panel, lane 8 compared with lane 4, quantified in Fig. 3H) to induce annexin A1 secretion in LPS-stimulated BMDMs while not changing lysate annexin A1 (Supplemental Fig. 3E, 3F). As knockdown using siRNA did not completely abolish NRF2 (Fig. 3E, quantified in Supplemental Fig. 3G), the hypothesis was also tested in BMDMs isolated from NRF2 knockout mice and matched wild-types. In NRF2 knockout macrophages stimulated with LPS and 4-OI or DMF, annexin A1 was completely absent from the supernatants (Fig. 3I, bottom panel, lanes 11 and 12 compared with lanes 5 and 6, quantified in Fig. 3J, Supplemental Fig. 3I, 3J). Collectively, these data demonstrate that 4-OI and DMF induce annexin A1 secretion in an NRF2-dependent manner. Given that NRF2 is a potent transcription factor, we hypothesized that the increases in secretion may be mediated by increased transcription and translation of annexin A1 downstream of NRF2. Surprisingly, however, we found that although LPS alone induces transcription of annexin A1 after 6 h, this effect was blocked by 4-OI and DMF (Supplemental Fig. 4A, 4B), despite strong NRF2 activation and anti-inflammatory signaling seen by induction of NRF2 target gene *Hmox1* and blocking of *Il1b* transcription (Supplemental Fig. 4C–F). These data highlight that 4-OI and DMF induce secretion independent of changes in the transcription of annexin A1.

### 4-OI and DMF induce annexin A1 secretion in an ABCA1-dependent manner

**FIGURE 4.**
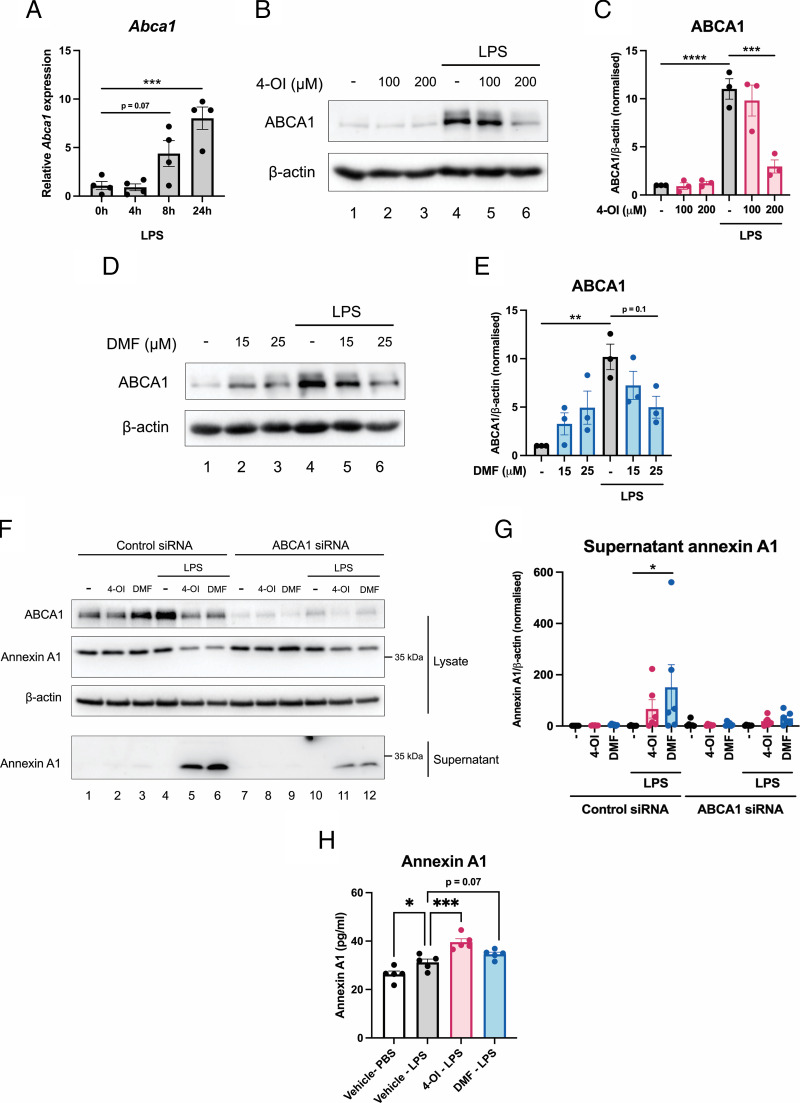
4-OI– and DMF-induced annexin A1 secretion requires ABCA1. (**A**) BMDMs were treated with LPS (100 ng/ml) for 4, 8, or 24 h. The cells were lysed, mRNA was extracted, and the expression of *Abca1* was quantified by quantitative PCR (*n* = 4 from three independent experiments). (**B**) Representative Western blots from BMDMs pretreated with vehicle or 100 or 200 μM 4-OI for 2 h prior to stimulation with LPS (100 ng/ml) for 24 h and cell lysates were harvested. (**C**) ABCA1 was measured by Western blotting (*n* = 3 from three independent experiments). (**D**) Representative Western blots from BMDMs pretreated with vehicle or 15 or 25 μM DMF for 2 h prior to stimulation with LPS (100 ng/ml) for 24 h and cell lysates were harvested. (**E**) ABCA1 was measured by Western blotting (*n* = 3 from three independent experiments), and quantification by densitometry is shown. (**F**) Representative Western blots from BMDMs that were transfected with 50 nM control siRNA or ABCA1 siRNA for 24 h. The cells were then pretreated with 200 μM 4-OI or 25 μM DMF for 2 h prior to stimulation with LPS (100 ng/ml) for 24 h. (**G**) Quantification of supernatant annexin A1 (*n* = 6 from three independent experiments). (**H**) Serum annexin A1 from mice injected with vehicle, 50 mg/kg 4-OI, or 25 mg/kg DMF 2 h prior to injection with PBS or LPS (2.5 mg/kg) as indicated (*n* = 4–5 per group). Data are presented as mean ± SEM, and a one-way ANOVA was performed. The data show the adjusted *p* value obtained from multiple comparisons, corrected for using the Tukey test. *p < 0.05, **p < 0.005, ***p < 0.0005, ****p < 0.0001.

Given that we have only seen an effect of the secretion of annexin A1, and not transcription, we next wondered which transporters could be involved in this process. The cholesterol transporter ABCA1 has been implicated in the secretion of annexin A1 (22, 23). Additionally, consistent with anti-inflammatory actions of 4-OI and DMF, ABCA1 has also been reported to reduce the macrophage inflammatory response to LPS potentially by regulating cell surface TLR4 (49) and to activate a Jak2-Stat3–dependent pathway to suppress IL-1β, IL-6, and TNF-α in response to LPS (24). Therefore, we assessed the possible involvement of ABCA1 in 4-OI– and DMF-induced annexin A1 secretion. We found that LPS increased mRNA levels of *Abca1* (Fig. 4A), which was previously reported in the literature (50). We hypothesized that perhaps NRF2 activation could be connected to secretion of annexin A1 via ABCA1 induction. There are reports of NRF2-activating compounds increasing ABCA1 expression in macrophages (51, 52), so we hypothesized that NRF2 activation by 4-OI and DMF led to an upregulation of ABCA1 expression, which would facilitate annexin A1 release. We analyzed ABCA1 expression by Western blotting and found that LPS also increased protein levels of ABCA1 (Fig. 4B, lane 4 compared with lane 1, quantified in Fig. 4C). However, contrary to our hypothesis, 4-OI did not augment ABCA1 expression, but in fact reduced LPS-induced ABCA1 expression (Fig. 4B, lane 6 compared with lane 4, quantified in Fig. 4C). Similar results were obtained in cells treated with DMF, with DMF tending to attenuate LPS-induced ABCA1 expression (Fig. 4F, lane 6 compared with lane 4, quantified in Fig. 4G). Although ABCA1 expression was not upregulated, but actually decreased by 4-OI and DMF, we still wanted to evaluate whether ABCA1 was required for this process. Although annexin A1 secretion in other cell types has been shown to be dependent on ABCA1 (22, 23), this has not been explicitly demonstrated for macrophages. When we knocked down ABCA1 using siRNA (Fig. 4H, quantified in Supplemental Fig. 4G), 4-OI– and DMF-induced annexin A1 secretion was markedly impaired whereas lysate annexin A1 was not changed (Fig. 4F, bottom panel, lanes 11 and 12 compared with lanes 5 and 6, quantified in Fig. 4G, Supplemental Fig. 4H), suggesting that secretion of annexin A1 from macrophages is ABCA1-dependent. Finally, we wanted to determine whether this system was also functional in vivo. Therefore, using an LPS sepsis model with 4-OI or DMF pretreatment, we found that LPS alone significantly increased annexin A1 in the serum and this was further boosted by 4-OI pretreatment, with a similar trend seen with DMF (*p* = 0.07) (Fig. 4H). Taken together, these data highlight that 4-OI and DMF induce annexin A1 secretion through a transporter mechanism rather than expression/transcription both in vitro and in vivo.

## Discussion

In recent years there has been a strong focus on elucidating the anti-inflammatory effects of the itaconate and fumarate derivatives 4-OI and DMF. These two Krebs cycle metabolite derivatives overlap in their effects, including attenuation of pyroptosis (38, 45), negative regulation of glycolysis (40, 43), downregulation of PGs (53), and, importantly, activation of NRF2 (27, 36). The findings of the current study now also suggest that 4-OI and DMF promote annexin A1 secretion. We demonstrate that both 4-OI and DMF, but not endogenous itaconate and fumarate, induce the secretion of a 33-kDa form of annexin A1 from macrophages, an effect that is far more striking in cells stimulated with the TLR4 agonist LPS. We show that additional ways of activating NRF2 through both pharmacological and genetic means are sufficient to drive annexin A1 secretion and that 4-OI and DMF cannot induce annexin A1 secretion in NRF2-deficient cells. We also provide evidence indicating that the transporter ABCA1 is required for secretion of annexin A1 in response to 4-OI and DMF. However, further work is necessary to understand exactly how NRF2 activation leads to annexin A1 secretion.

We hypothesized that perhaps NRF2 activation by 4-OI and DMF boosted ABCA1 expression, as other NRF2 activators have been reported to do so (51, 52), although these studies were primarily concerned with the facilitation of cholesterol efflux by ABCA1. Our data suggest that the mechanism by which NRF2 activation gives rise to annexin A1 secretion is not through upregulation of ABCA1 because 4-OI and DMF did not augment its expression and, if anything, were inhibitory. However, ABCA1 is sensitive to palmitoylation on several cysteine residues for its cellular localization to the plasma membrane, and for its export function (54). Therefore, it is possible that DMF and 4-OI are either directly targeting cysteine residues to alter palmitoylation or modifying other targets involved in ABCA1’s regulation and function such as the palmitoyl transferases, which have been shown to be targeted by cysteine-reactive compounds (44). Furthermore, whether NRF2 activation drives transcription of these regulators of ABCA1 is currently unknown. Additionally, that LPS increases ABCA1 expression might serve as a primer for 4-OI and DMF to then modify activity or activity-modulating enzymes to induce annexin A1 secretion. This might be why these effects are much stronger in combination with LPS stimulation in macrophages than when 4-OI or DMF are used alone.

Although it has been known for some time that annexin A1 is secreted, the regulation of this process remains poorly understood. It is striking that the 33-kDa cleaved form of annexin A1 that was consistently detected in the supernatant was not detected in the cell lysates. Perhaps this indicates that the cleavage event somehow drives the secretion of annexin A1. It was demonstrated for neutrophils that proteinase 3 and elastase can cleave annexin A1 to the 33-kDa form (25, 26). Perhaps NRF2 activation drives expression or activation of one of these proteases, or alternative proteases that are yet to be implicated in annexin A1 cleavage, and in this way induces annexin A1 cleavage and subsequent secretion. However, investigation into the identity and mechanism of the protease responsible for annexin A1 cleavage in response to NRF2 activation is outside the scope of this study.

These data also emphasize that metabolite derivatives do not always mimic the action of their corresponding endogenous metabolites, as recently reviewed (34). Many of the effects that have been shown for 4-OI have also been found to be recapitulated by endogenous itaconate, which is usually studied using *Irg1*^−/−^ mice. For example, both 4-OI and endogenous itaconate have been shown to limit glycolysis (40), inhibit TET DNA dioxygenases (42), and induce lysosomal biogenesis (55) in macrophages. However, increasing evidence has emerged that 4-OI and itaconate sometimes diverge in their effects. 4-OI has been reported to downregulate type I IFNs (27) whereas itaconate was found to boost IFN production (56). We have previously reported that 4-OI reduced PG production in macrophages yet endogenous itaconate did not (53), and in this study again we see that endogenous itaconate and fumarate fail to recapitulate the effects of the derivatives 4-OI and DMF. One potential explanation for this could be the differing degrees to which 4-OI and endogenous itaconate activate NRF2. Although it is known that 4-OI potently activates NRF2 (27), there are some discrepancies in the literature regarding NRF2 activation by itaconate. One study showed that LPS-induced NRF2 levels were lower in *Irg1*^−/−^ BMDMs compared with wild-type cells (47). However, a later study from the same group showed no changes in NRF2 activation with exogenous itaconate up to 7.5 mM (56), and another group published that there was no difference in LPS-induced NRF2 stabilization between *Irg1^+/+^* and *Irg1*^−/−^ BMDMs (57). Therefore, the extent to which endogenous itaconate can activate NRF2 is unclear and could explain why endogenous itaconate does not affect annexin A1 secretion, which our data suggest is an NRF2-dependent process. Perhaps a threshold of NRF2 activation is required, which LPS alone cannot achieve, even though it induces itaconate accumulation. 4-OI might breach that threshold, promoting strong NRF2 activation and annexin A1 secretion.

Annexin A1 has actually been reported to be modified by itaconate and itaconate derivatives in multiple studies (27, 41, 44, 58), yet the functional consequence of this modification has not been thus far elucidated. Intriguingly, the same cysteine residue on annexin A1, Cys^189^, has been shown to be modified by DMF in neurons and astrocytes (59), and low annexin A1 levels were found to correlate with severity of relapsing-remitting MS (60), a disease for which DMF is used as treatment. It is possible that 4-OI and DMF might drive annexin A1 secretion through a combination of NRF2 activation, direct modification of annexin A1, and regulation of ABCA1 activity. It is conceivable, for example, that NRF2 activation drives annexin A1 cleavage while the modification is required for its translocation to the plasma membrane for secretion, as it has already been reported that annexin A1 phosphorylation regulates trafficking of annexin A1 to the plasma membrane (61, 62). As the modification of NLRP3 by 4-OI was shown to modulate the direct interaction between NLRP3 and NEK7 (38), the modification of annexin A1 could potentially regulate its interaction with ABCA1. It is also feasible that the modification could modulate binding of annexin A1 to its receptor FPR2 after it has been secreted. As our data show NRF2 dependency, modification of annexin A1 by 4-OI or DMF alone is not sufficient to induce secretion. The potential downstream effects of annexin A1 modification therefore require further analysis.

Importantly, this work highlights, to our knowledge for the first time, that the cysteine-reactive compounds 4-OI and DMF induce the secretion of the potent proresolution molecule annexin A1 both in vitro and in vivo, and that this effect requires both NRF2 and ABCA1. Although some gaps remain in our knowledge of how 4-OI and DMF drive annexin A1 secretion in LPS-stimulated macrophages and mice, our data nonetheless contribute to the already substantial body of literature detailing 4-OI and DMF as potent anti-inflammatory agents. Finally, these data provide evidence that clinically used therapeutic DMF could be used to promote resolution via annexin A1 in other inflammatory conditions, and that other cysteine-reactive compounds, such as 4-OI, could similarly be used to promote resolution of inflammation.

## Supplementary Material

Supplemental 1 (PDF)

## References

[1] De Caterina, R., R. Sicari, D. Giannessi, P. L. Paggiaro, P. Paoletti, G. Lazzerini, W. Bernini, E. Solito, L. Parente. 1993. Macrophage-specific eicosanoid synthesis inhibition and lipocortin-1 induction by glucocorticoids. J Appl Physiol (1985) 75: 2368–2375. 8125852 10.1152/jappl.1993.75.6.2368

[2] Solito, E., C. Raguenes-Nicol, C. de Coupade, A. Bisagni-Faure, F. Russo-Marie. 1998. U937 cells deprived of endogenous annexin 1 demonstrate an increased PLA2 activity. Br. J. Pharmacol. 124: 1675–1683. 9756383 10.1038/sj.bjp.0701991 PMC1565558

[3] Kim, K. M., D. K. Kim, Y. M. Park, C. K. Kim, D. S. Na. 1994. Annexin-I inhibits phospholipase A_2_ by specific interaction, not by substrate depletion. FEBS Lett. 343: 251–255. 8174710 10.1016/0014-5793(94)80566-0

[4] Chatterjee, B. E., S. Yona, G. Rosignoli, R. E. Young, S. Nourshargh, R. J. Flower, M. Perretti. 2005. Annexin 1-deficient neutrophils exhibit enhanced transmigration in vivo and increased responsiveness in vitro. J. Leukoc. Biol. 78: 639–646. 16000391 10.1189/jlb.0405206

[5] Hayhoe, R. P., A. M. Kamal, E. Solito, R. J. Flower, D. Cooper, M. Perretti. 2006. Annexin 1 and its bioactive peptide inhibit neutrophil-endothelium interactions under flow: indication of distinct receptor involvement. Blood 107: 2123–2130. 16278303 10.1182/blood-2005-08-3099

[6] Solito, E., A. Kamal, F. Russo-Marie, J. C. Buckingham, S. Marullo, M. Perretti. 2003. A novel calcium-dependent proapoptotic effect of annexin 1 on human neutrophils. FASEB J. 17: 1544–1546. 12824302 10.1096/fj.02-0941fje

[7] Sinniah, A., S. Yazid, S. Bena, S. M. Oliani, M. Perretti, R. J. Flower. 2019. Endogenous annexin-A1 negatively regulates mast cell-mediated allergic reactions. Front. Pharmacol. 10: 1313. 31798445 10.3389/fphar.2019.01313 PMC6865276

[8] Parisi, J. D. S., M. P. Corrêa, C. D. Gil. 2019. Lack of endogenous annexin A1 increases mast cell activation and exacerbates experimental atopic dermatitis. Cells 8: 51. 30650525 10.3390/cells8010051 PMC6356645

[9] Oliveira, M. P., J. Prates, A. D. Gimenes, S. G. Correa, S. M. Oliani. 2021. Annexin A1 mimetic peptide Ac_2-26_ modulates the function of murine colonic and human mast cells. Front. Immunol. 12: 689484. 34557187 10.3389/fimmu.2021.689484 PMC8452975

[10] Ferlazzo, V., P. D’Agostino, S. Milano, R. Caruso, S. Feo, E. Cillari, L. Parente. 2003. Anti-inflammatory effects of annexin-1: stimulation of IL-10 release and inhibition of nitric oxide synthesis. Int. Immunopharmacol. 3: 1363–1369. 12946433 10.1016/S1567-5769(03)00133-4

[11] Scannell, M., M. B. Flanagan, A. deStefani, K. J. Wynne, G. Cagney, C. Godson, P. Maderna. 2007. Annexin-1 and peptide derivatives are released by apoptotic cells and stimulate phagocytosis of apoptotic neutrophils by macrophages. J. Immunol. 178: 4595–4605. 17372018 10.4049/jimmunol.178.7.4595

[12] McArthur, S., G. Juban, T. Gobbetti, T. Desgeorges, M. Theret, J. Gondin, J. E. Toller-Kawahisa, C. P. Reutelingsperger, B. Chazaud, M. Perretti, R. Mounier. 2020. Annexin A1 drives macrophage skewing to accelerate muscle regeneration through AMPK activation. J. Clin. Invest. 130: 1156–1167. 32015229 10.1172/JCI124635 PMC7269594

[13] Xu, X., W. Gao, L. Li, J. Hao, B. Yang, T. Wang, L. Li, X. Bai, F. Li, H. Ren, . 2021. Annexin A1 protects against cerebral ischemia-reperfusion injury by modulating microglia/macrophage polarization via FPR2/ALX-dependent AMPK-mTOR pathway. J. Neuroinflammation 18: 119. 34022892 10.1186/s12974-021-02174-3 PMC8140477

[14] Day, E. A., R. J. Ford, G. R. Steinberg. 2017. AMPK as a therapeutic target for treating metabolic diseases. Trends Endocrinol. Metab. 28: 545–560. 28647324 10.1016/j.tem.2017.05.004

[15] Galic, S., M. D. Fullerton, J. D. Schertzer, S. Sikkema, K. Marcinko, C. R. Walkley, D. Izon, J. Honeyman, Z. P. Chen, B. J. van Denderen, . 2011. Hematopoietic AMPK β1 reduces mouse adipose tissue macrophage inflammation and insulin resistance in obesity. J. Clin. Invest. 121: 4903–4915. 22080866 10.1172/JCI58577 PMC3226000

[16] O’Neill, L. A., D. G. Hardie. 2013. Metabolism of inflammation limited by AMPK and pseudo-starvation. Nature 493: 346–355. 23325217 10.1038/nature11862

[17] Mounier, R., M. Théret, L. Arnold, S. Cuvellier, L. Bultot, O. Göransson, N. Sanz, A. Ferry, K. Sakamoto, M. Foretz, . 2013. AMPKα1 regulates macrophage skewing at the time of resolution of inflammation during skeletal muscle regeneration. Cell Metab. 18: 251–264. 23931756 10.1016/j.cmet.2013.06.017

[18] Purvis, G. S. D., E. Solito, C. Thiemermann. 2019. Annexin-A1: therapeutic potential in microvascular disease. Front. Immunol. 10: 938. 31114582 10.3389/fimmu.2019.00938 PMC6502989

[19] Christmas, P., J. Callaway, J. Fallon, J. Jones, H. T. Haigler. 1991. Selective secretion of annexin 1, a protein without a signal sequence, by the human prostate gland. J. Biol. Chem. 266: 2499–2507. 1824943

[20] Tall, A. R., P. Costet, N. Wang. 2002. Regulation and mechanisms of macrophage cholesterol efflux. J. Clin. Invest. 110: 899–904. 12370265 10.1172/JCI16391 PMC151157

[21] Wein, S., M. Fauroux, J. Laffitte, P. de Nadaï, C. Guaïni, F. Pons, C. Coméra. 2004. Mediation of annexin 1 secretion by a probenecid-sensitive ABC-transporter in rat inflamed mucosa. Biochem. Pharmacol. 67: 1195–1202. 15006554 10.1016/j.bcp.2003.11.015

[22] Omer, S., D. Meredith, J. F. Morris, H. C. Christian. 2006. Evidence for the role of adenosine 5′-triphosphate-binding cassette (ABC)-A1 in the externalization of annexin 1 from pituitary folliculostellate cells and ABCA1-transfected cell models. Endocrinology 147: 3219–3227. 16601136 10.1210/en.2006-0099

[23] Li, L., L. Xu, W. Chen, X. Li, Q. Xia, L. Zheng, Q. Duan, H. Zhang, Y. Zhao. 2018. Reduced annexin A1 secretion by ABCA1 causes retinal inflammation and ganglion cell apoptosis in a murine glaucoma model. Front. Cell. Neurosci. 12: 347. 30364320 10.3389/fncel.2018.00347 PMC6193130

[24] Tang, C., Y. Liu, P. S. Kessler, A. M. Vaughan, J. F. Oram. 2009. The macrophage cholesterol exporter ABCA1 functions as an anti-inflammatory receptor. J. Biol. Chem. 284: 32336–32343. 19783654 10.1074/jbc.M109.047472 PMC2781648

[25] Rescher, U., V. Goebeler, A. Wilbers, V. Gerke. 2006. Proteolytic cleavage of annexin 1 by human leukocyte elastase. Biochim. Biophys. Acta 1763: 1320–1324. 17023068 10.1016/j.bbamcr.2006.08.041

[26] Vong, L., F. D’Acquisto, M. Pederzoli-Ribeil, L. Lavagno, R. J. Flower, V. Witko-Sarsat, M. Perretti. 2007. Annexin 1 cleavage in activated neutrophils: a pivotal role for proteinase 3. J. Biol. Chem. 282: 29998–30004. 17681950 10.1074/jbc.M702876200 PMC2772024

[27] Mills, E. L., D. G. Ryan, H. A. Prag, D. Dikovskaya, D. Menon, Z. Zaslona, M. P. Jedrychowski, A. S. H. Costa, M. Higgins, E. Hams, . 2018. Itaconate is an anti-inflammatory metabolite that activates Nrf2 via alkylation of KEAP1. Nature 556: 113–117. 29590092 10.1038/nature25986 PMC6047741

[28] Hooftman, A., C. G. Peace, D. G. Ryan, E. A. Day, M. Yang, A. F. McGettrick, M. Yin, E. N. Montano, L. Huo, J. E. Toller-Kawahisa, . 2023. Macrophage fumarate hydratase restrains mtRNA-mediated interferon production. Nature 615: 490–498. 36890227 10.1038/s41586-019-0000-0 PMC10411300

[29] Lee, C. G., N. A. Jenkins, D. J. Gilbert, N. G. Copeland, W. E. O’Brien. 1995. Cloning and analysis of gene regulation of a novel LPS-inducible cDNA. Immunogenetics 41: 263–270. 7721348 10.1007/BF00172150

[30] Michelucci, A., T. Cordes, J. Ghelfi, A. Pailot, N. Reiling, O. Goldmann, T. Binz, A. Wegner, A. Tallam, A. Rausell, . 2013. Immune-responsive gene 1 protein links metabolism to immunity by catalyzing itaconic acid production. Proc. Natl. Acad. Sci. USA 110: 7820–7825. 23610393 10.1073/pnas.1218599110 PMC3651434

[31] McFadden, B. A., S. Purohit. 1977. Itaconate, an isocitrate lyase-directed inhibitor in *Pseudomonas indigofera*. J. Bacteriol. 131: 136–144. 17593 10.1128/jb.131.1.136-144.1977 PMC235402

[32] Naujoks, J., C. Tabeling, B. D. Dill, C. Hoffmann, A. S. Brown, M. Kunze, S. Kempa, A. Peter, H. J. Mollenkopf, A. Dorhoi, . 2016. IFNs modify the proteome of *Legionella*-containing vacuoles and restrict infection via IRG1-derived itaconic acid. PLoS Pathog. 12: e1005408. 26829557 10.1371/journal.ppat.1005408 PMC4734697

[33] Hooftman, A., L. A. J. O’Neill. 2019. The immunomodulatory potential of the metabolite itaconate. Trends Immunol. 40: 687–698. 31178405 10.1016/j.it.2019.05.007

[34] Day, E. A., L. A. J. O’Neill. 2022. Protein targeting by the itaconate family in immunity and inflammation. Biochem. J. 479: 2499–2510. 36546613 10.1042/BCJ20220364

[35] Alderson, N. L., Y. Wang, M. Blatnik, N. Frizzell, M. D. Walla, T. J. Lyons, N. Alt, J. A. Carson, R. Nagai, S. R. Thorpe, J. W. Baynes. 2006. *S*-(2-Succinyl)cysteine: a novel chemical modification of tissue proteins by a Krebs cycle intermediate. Arch. Biochem. Biophys. 450: 1–8. 16624247 10.1016/j.abb.2006.03.005

[36] Linker, R. A., D. H. Lee, S. Ryan, A. M. van Dam, R. Conrad, P. Bista, W. Zeng, X. Hronowsky, A. Buko, S. Chollate, . 2011. Fumaric acid esters exert neuroprotective effects in neuroinflammation via activation of the Nrf2 antioxidant pathway. Brain 134: 678–692. 21354971 10.1093/brain/awq386

[37] Kobayashi, E. H., T. Suzuki, R. Funayama, T. Nagashima, M. Hayashi, H. Sekine, N. Tanaka, T. Moriguchi, H. Motohashi, K. Nakayama, M. Yamamoto. 2016. Nrf2 suppresses macrophage inflammatory response by blocking proinflammatory cytokine transcription. Nat. Commun. 7: 11624. 27211851 10.1038/ncomms11624 PMC4879264

[38] Hooftman, A., S. Angiari, S. Hester, S. E. Corcoran, M. C. Runtsch, C. Ling, M. C. Ruzek, P. F. Slivka, A. F. McGettrick, K. Banahan, . 2020. The immunomodulatory metabolite itaconate modifies NLRP3 and inhibits inflammasome activation. Cell Metab. 32: 468–478.e7. 32791101 10.1016/j.cmet.2020.07.016 PMC7422798

[39] Bambouskova, M., L. Potuckova, T. Paulenda, M. Kerndl, D. A. Mogilenko, K. Lizotte, A. Swain, S. Hayes, R. D. Sheldon, H. Kim, . 2021. Itaconate confers tolerance to late NLRP3 inflammasome activation. Cell Rep. 34: 108756. 33691097 10.1016/j.celrep.2021.108756 PMC8039864

[40] Liao, S. T., C. Han, D. Q. Xu, X. W. Fu, J. S. Wang, L. Y. Kong. 2019. 4-Octyl itaconate inhibits aerobic glycolysis by targeting GAPDH to exert anti-inflammatory effects. Nat. Commun. 10: 5091. 31704924 10.1038/s41467-019-13078-5 PMC6841710

[41] Qin, W., K. Qin, Y. Zhang, W. Jia, Y. Chen, B. Cheng, L. Peng, N. Chen, Y. Liu, W. Zhou, . 2019. *S*-glycosylation-based cysteine profiling reveals regulation of glycolysis by itaconate. Nat. Chem. Biol. 15: 983–991. 31332308 10.1038/s41589-019-0323-5

[42] Chen, L. L., C. Morcelle, Z. L. Cheng, X. Chen, Y. Xu, Y. Gao, J. Song, Z. Li, M. D. Smith, M. Shi, . 2022. Itaconate inhibits TET DNA dioxygenases to dampen inflammatory responses. Nat. Cell Biol. 24: 353–363. 35256775 10.1038/s41556-022-00853-8 PMC9305987

[43] Kornberg, M. D., P. Bhargava, P. M. Kim, V. Putluri, A. M. Snowman, N. Putluri, P. A. Calabresi, S. H. Snyder. 2018. Dimethyl fumarate targets GAPDH and aerobic glycolysis to modulate immunity. Science 360: 449–453. 29599194 10.1126/science.aan4665 PMC5924419

[44] Qin, W., Y. Zhang, H. Tang, D. Liu, Y. Chen, Y. Liu, C. Wang. 2020. Chemoproteomic profiling of itaconation by bioorthogonal probes in inflammatory macrophages. J. Am. Chem. Soc. 142: 10894–10898. 32496768 10.1021/jacs.9b11962

[45] Humphries, F., L. Shmuel-Galia, N. Ketelut-Carneiro, S. Li, B. Wang, V. V. Nemmara, R. Wilson, Z. Jiang, F. Khalighinejad, K. Muneeruddin, . 2020. Succination inactivates gasdermin D and blocks pyroptosis. Science 369: 1633–1637. 32820063 10.1126/science.abb9818 PMC8744141

[46] Linker, R. A., A. Haghikia. 2016. Dimethyl fumarate in multiple sclerosis: latest developments, evidence and place in therapy. Ther. Adv. Chronic Dis. 7: 198–207. 27433310 10.1177/2040622316653307 PMC4935836

[47] Bambouskova, M., L. Gorvel, V. Lampropoulou, A. Sergushichev, E. Loginicheva, K. Johnson, D. Korenfeld, M. E. Mathyer, H. Kim, L. H. Huang, . 2018. Electrophilic properties of itaconate and derivatives regulate the IκBζ-ATF3 inflammatory axis. Nature 556: 501–504. 29670287 10.1038/s41586-018-0052-z PMC6037913

[48] Kobayashi, M., L. Li, N. Iwamoto, Y. Nakajima-Takagi, H. Kaneko, Y. Nakayama, M. Eguchi, Y. Wada, Y. Kumagai, M. Yamamoto. 2009. The antioxidant defense system Keap1-Nrf2 comprises a multiple sensing mechanism for responding to a wide range of chemical compounds. Mol. Cell. Biol. 29: 493–502. 19001094 10.1128/MCB.01080-08 PMC2612520

[49] Yvan-Charvet, L., C. Welch, T. A. Pagler, M. Ranalletta, M. Lamkanfi, S. Han, M. Ishibashi, R. Li, N. Wang, A. R. Tall. 2008. Increased inflammatory gene expression in ABC transporter-deficient macrophages: free cholesterol accumulation, increased signaling via Toll-like receptors, and neutrophil infiltration of atherosclerotic lesions. Circulation 118: 1837–1847. 18852364 10.1161/CIRCULATIONAHA.108.793869 PMC2756536

[50] Kaplan, R., X. Gan, J. G. Menke, S. D. Wright, T. Q. Cai. 2002. Bacterial lipopolysaccharide induces expression of ABCA1 but not ABCG1 via an LXR-independent pathway. J. Lipid Res. 43: 952–959. 12032171

[51] Lu, Q., S. L. Tang, X. Y. Liu, G. J. Zhao, X. P. Ouyang, Y. C. Lv, P. P. He, F. Yao, W. J. Chen, Y. Y. Tang, . 2013. Tertiary-butylhydroquinone upregulates expression of ATP-binding cassette transporter A1 via nuclear factor E2-related factor 2/heme oxygenase-1 signaling in THP-1 macrophage-derived foam cells. Circ. J. 77: 2399–2408. 23739547 10.1253/circj.cj-12-1616

[52] Zhong, Y., J. Feng, Z. Fan, J. Li. 2018. Curcumin increases cholesterol efflux via heme oxygenase-1-mediated ABCA1 and SR-BI expression in macrophages. Mol. Med. Rep. 17: 6138–6143. 29436680 10.3892/mmr.2018.8577

[53] Diskin, C., A. Zotta, S. E. Corcoran, V. J. Tyrrell, Z. Zaslona, V. B. O’Donnell, L. A. J. O’Neill. 2021. 4-Octyl-itaconate and dimethyl fumarate inhibit COX2 expression and prostaglandin production in macrophages. J. Immunol. 207: 2561–2569. 34635585 10.4049/jimmunol.2100488 PMC7613254

[54] Singaraja, R. R., M. H. Kang, K. Vaid, S. S. Sanders, G. L. Vilas, P. Arstikaitis, J. Coutinho, R. C. Drisdel, A. D. El-Husseini, W. N. Green, . 2009. Palmitoylation of ATP-binding cassette transporter A1 is essential for its trafficking and function. Circ. Res. 105: 138–147. 19556522 10.1161/CIRCRESAHA.108.193011

[55] Zhang, Z., C. Chen, F. Yang, Y. X. Zeng, P. Sun, P. Liu, X. Li. 2022. Itaconate is a lysosomal inducer that promotes antibacterial innate immunity. Mol. Cell 82: 2844–2857.e10. 35662396 10.1016/j.molcel.2022.05.009

[56] Swain, A., M. Bambouskova, H. Kim, P. S. Andhey, D. Duncan, K. Auclair, V. Chubukov, D. M. Simons, T. P. Roddy, K. M. Stewart, M. N. Artyomov. 2020. Comparative evaluation of itaconate and its derivatives reveals divergent inflammasome and type I interferon regulation in macrophages. Nat. Metab. 2: 594–602. 32694786 10.1038/s42255-020-0210-0 PMC7378276

[57] Sun, K. A., Y. Li, A. Y. Meliton, P. S. Woods, L. M. Kimmig, R. Cetin-Atalay, R. B. Hamanaka, G. M. Mutlu. 2020. Endogenous itaconate is not required for particulate matter-induced NRF2 expression or inflammatory response. eLife 9: e54877. 32255424 10.7554/eLife.54877 PMC7185992

[58] Cifani, P., Z. Li, D. Luo, M. Grivainis, A. M. Intlekofer, D. Fenyö, A. Kentsis. 2021. Discovery of protein modifications using differential tandem mass spectrometry proteomics. J. Proteome Res. 20: 1835–1848. 33749263 10.1021/acs.jproteome.0c00638 PMC8341206

[59] Piroli, G. G., A. M. Manuel, T. Patel, M. D. Walla, L. Shi, S. A. Lanci, J. Wang, A. Galloway, P. I. Ortinski, D. S. Smith, N. Frizzell. 2019. Identification of novel protein targets of dimethyl fumarate modification in neurons and astrocytes reveals actions independent of Nrf2 stabilization. Mol. Cell. Proteomics 18: 504–519. 30587509 10.1074/mcp.RA118.000922 PMC6398201

[60] Colamatteo, A., E. Maggioli, R. Azevedo Loiola, M. Hamid Sheikh, G. Calì, D. Bruzzese, G. T. Maniscalco, D. Centonze, F. Buttari, R. Lanzillo, . 2019. Reduced annexin A1 expression associates with disease severity and inflammation in multiple sclerosis patients. J. Immunol. 203: 1753–1765. 31462505 10.4049/jimmunol.1801683

[61] Solito, E., A. Mulla, J. F. Morris, H. C. Christian, R. J. Flower, J. C. Buckingham. 2003. Dexamethasone induces rapid serine-phosphorylation and membrane translocation of annexin 1 in a human folliculostellate cell line via a novel nongenomic mechanism involving the glucocorticoid receptor, protein kinase C, phosphatidylinositol 3-kinase, and mitogen-activated protein kinase. Endocrinology 144: 1164–1174. 12639897 10.1210/en.2002-220592

[62] Solito, E., H. C. Christian, M. Festa, A. Mulla, T. Tierney, R. J. Flower, J. C. Buckingham. 2006. Post-translational modification plays an essential role in the translocation of annexin A1 from the cytoplasm to the cell surface. FASEB J. 20: 1498–1500. 16720734 10.1096/fj.05-5319fje PMC2049060

