# Supplementary material for: 4-Octyl Itaconate and Dimethyl Fumarate Induce Secretion of the Anti-Inflammatory Protein Annexin A1 via NRF2
Source: J Immunol. 2023 Aug 14;211(6):1032–41. doi: 10.4049/jimmunol.2200848 (PMC10476164; doi:10.4049/jimmunol.2200848)
Supplement: Supplemental 1 (PDF) [file JI_2200848_Supplemental_1.pdf]

Supplemental Figures:

Supplemental Figure 1:

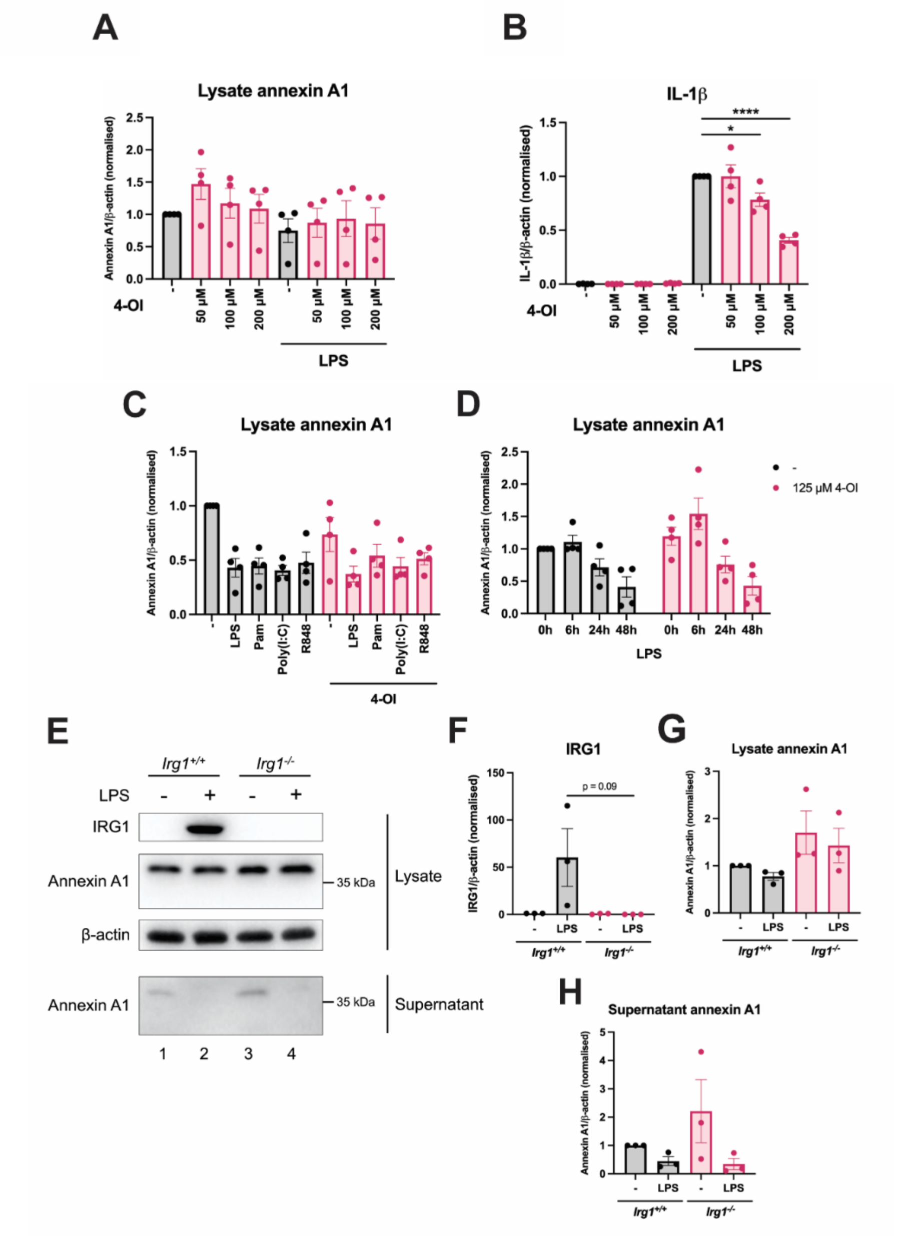

Supplemental Figure 1: 4-OI does not alter lysate annexin A1 in BMDMs. BMDMs were pretreated with 4-OI (50-200 μM) or vehicle for two hours prior to stimulation with LPS (100 ng/mL) for 24 hours. Cell lysates were harvested and cell supernatants were concentrated. Quantification of lysate **A**) annexin A1 and **B**) pro-IL-1β (*n* = 4 from three independent experiments). **C**) Lysate annexin A1 quantification from BMDMs pretreated with 200 μM 4-OI for two hours prior to stimulation with LPS (100 ng/mL), Pam3CSK4 (100 ng/mL), Poly (I:C) (1 μg/mL) or R848 (500 ng/mL) for 24 hours. (*n* = 3 from three independent experiments). **D)** Lysate annexin A1 quantification from BMDMs pretreated with 125 μM 4-OI for two hours prior to stimulation with LPS (100 ng/mL) for 6, 24 or 48 hours. (*n* = 4 from three independent experiments). **E**) Representative western blots from BMDMs from *Irg1*$_{+/+}$ and *Irg1*$_{-/-}$ mice were stimulated with LPS (100 ng/mL) for 24 hours. Cell lysates were harvested and cell supernatants were concentrated. Lysate **F**) IRG1 and **G**) annexin A1 and **H**) supernatant annexin A1, were measured by Western blotting, (*n* = 3 from three independent experiments). Data are presented as mean ± S.E.M and a one-way or two-way ANOVA was performed. The data show the adjusted p value obtained from multiple comparisons, corrected for using the Tukey test for one-way ANOVA or Šidák test for two-way ANOVA. *p < 0.05, ***p < 0.0005, ****p < 0.0001.

Supplemental Figure 2:

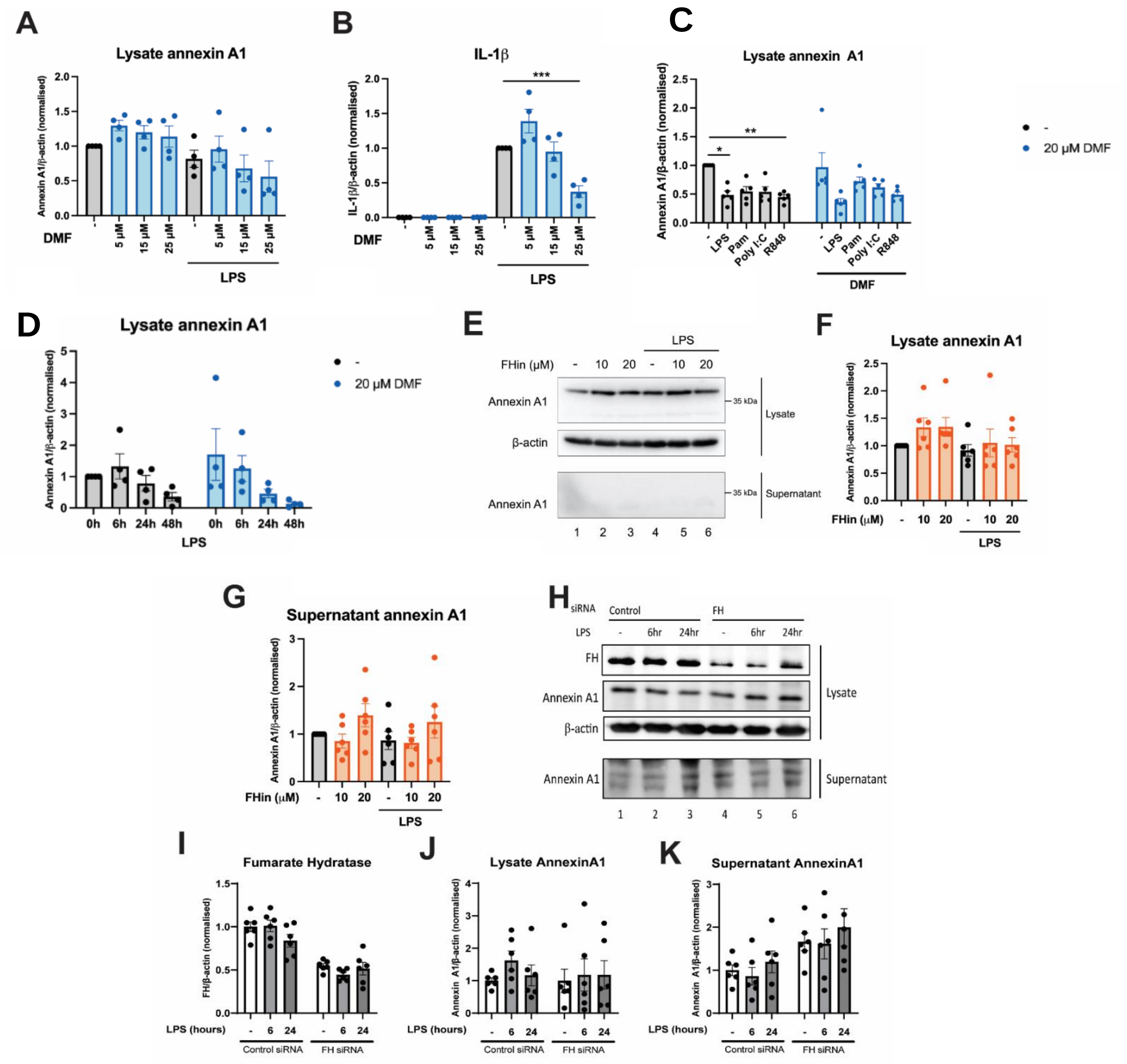

Supplemental Figure 2: DMF does not alter lysate annexin a1 in BMDMs. Lysate **A**) annexin A1 and **B**) pro-IL-1β from BMDMs were pretreated with DMF (5-25 µM) or vehicle for two hours prior to stimulation with LPS (100 ng/mL) for 24 hours. (*n* = 4 from three independent experiments). **C**) Quantification of lysate annexin A1 from BMDMs pretreated with 20 µM DMF for two hours prior to stimulation with LPS (100 ng/mL), Pam3CSK4 (100 ng/mL), Poly (I:C) (1 µg/mL) or R848 (500 ng/mL) for 24 hours. **D**) BMDMs were pretreated with 20 µM DMF for two hours prior to stimulation with LPS (100ng/mL) for 6, 24 or 48 hours and lysate annexin A1 was measured (*n* = 4 from three independent experiments). **E**) Representative western blots from BMDMs pretreated with FH inhibitor (10 or 20 µM) or vehicle for two hours prior to stimulation with LPS (100 ng/mL) for 24 hours. **F**) Lysate and **G**) supernatant annexin A1 levels were measured by Western blotting (*n* = 6 from three independent experiments) and quantification by densitometry is shown. **H**) Representative western blot from BMDMs transfected with control siRNA or FH siRNA and treated with LPS for 6 or 24hours. Quantification of **I**) fumarate hydratase, **J**) lysate annexin A1, and **K**) supernatant annexin A1(n= 5-6 from 3 independent experiments). Data are presented as mean ± S.E.M and a one-way or two-way ANOVA was performed. The data show the adjusted p value obtained from multiple comparisons, corrected for using the Tukey test for one-way ANOVA or Šidák test for two-way ANOVA. *p < 0.05, ***p < 0.0005

Supplemental Figure 3:

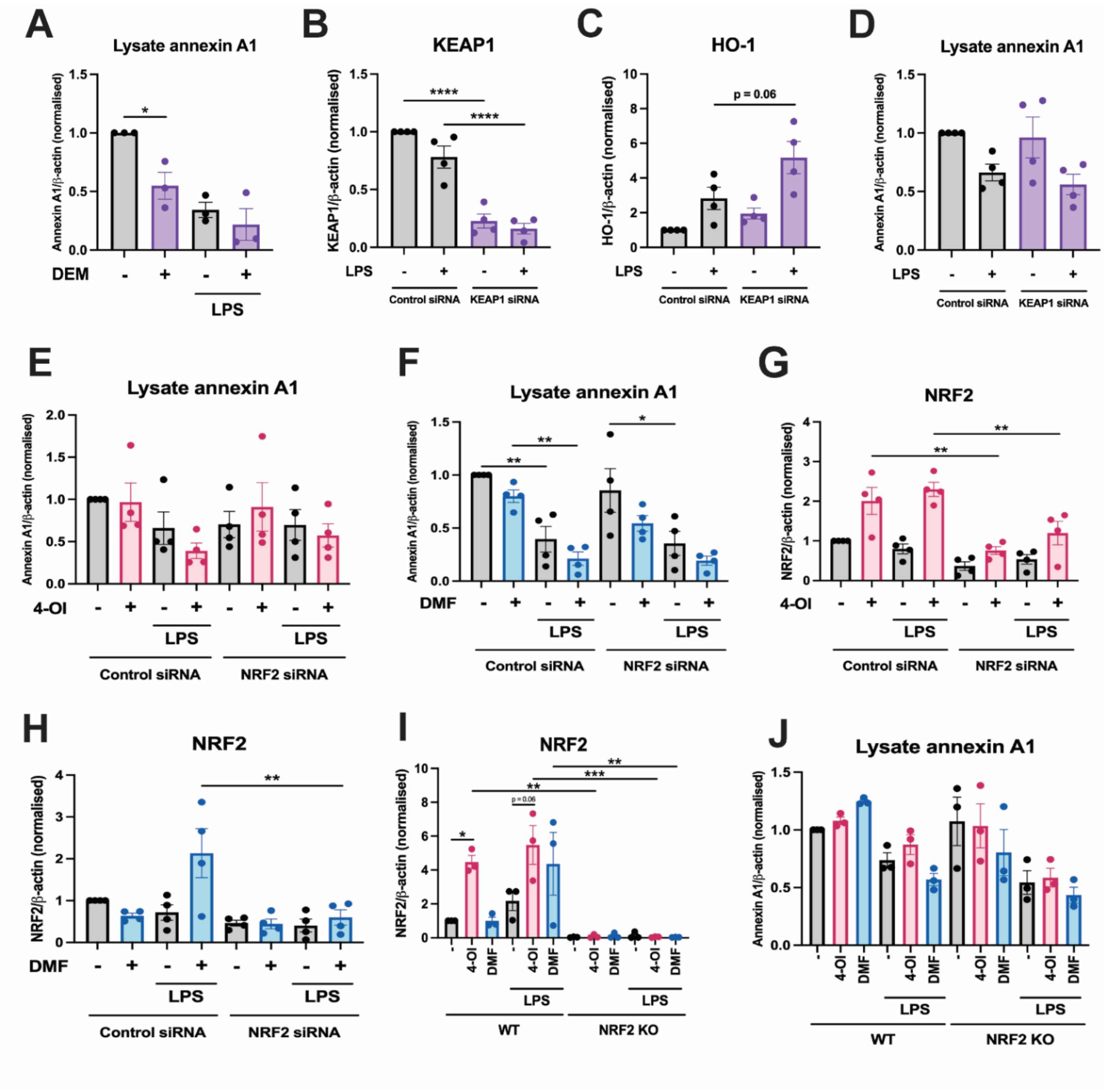

Supplemental Figure 3: 4-OI and DMF induce annexin A1 secretion in an NRF2-dependent manner. .**A**) Lysate annexin A1 levels were measured by Western blotting from BMDMs pretreated with 100 µM DEM for two hours prior to stimulation with LPS (100ng/mL) for 24 hours. (*n* = 3 from three independent experiments). Quantification of **B**) KEAP 1, **C**) HO-1 and **D**) Annexin A1 from BMDMs that were transfected with 50 nM control siRNA or KEAP1 siRNA for 48 hours, prior to stimulation with LPS (100 ng/mL) for 24 hours (*n* = 4 from three independent experiments). Quantification of lysate **E,F**) annexin A1 and **G,H**) NRF2 measured by Western blotting from BMDMs that were transfected with 50 nM control siRNA or NRF2 siRNA for 24 hours. The cells were then pretreated with 200µM 4-OI or 25µM DMF as indicated for two hours prior to stimulation with LPS (100 ng/mL) for 24 hours (*n* = 4 from three independent experiments. Lysate **I**) NRF2 and **J**) Annexin A1 from western blots of BMDMs from wild-type and NRF2 knockout mice were pretreated with vehicle, 200 µM 4-OI or 25 µM DMF for two hours prior to stimulation with LPS (100 ng/mL) for 24 hours. (*n* = 3 from one independent experiment). Data are presented as mean ± S.E.M and a one-way ANOVA was performed. The data show the adjusted p value obtained from multiple comparisons, corrected for using the Tukey test. *p < 0.05, **p < 0.005, ***p < 0.0005, ****p < 0.0001, ns = nonsignificant.

Supplemental Figure 4:

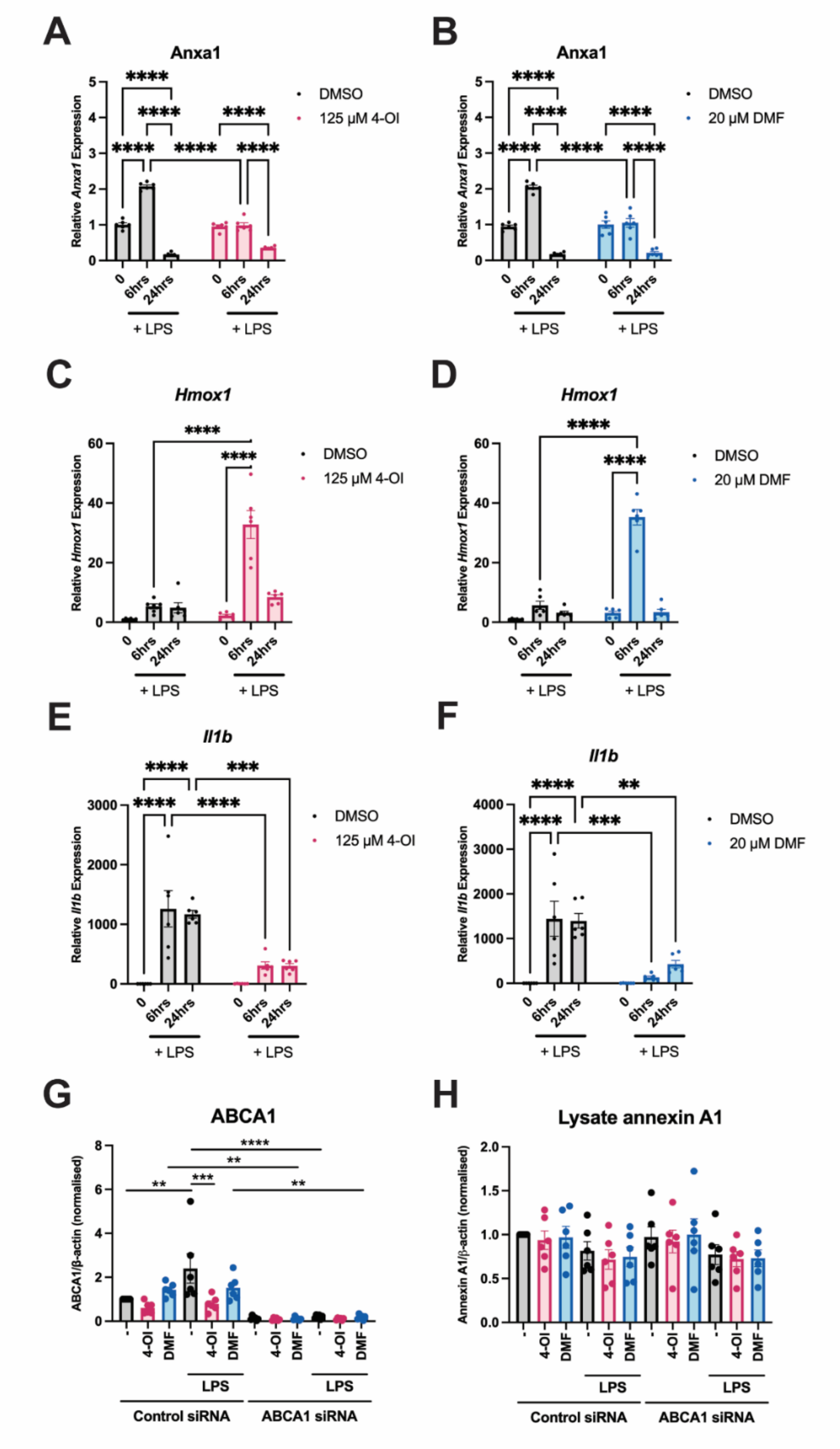

Supplemental Figure 4: **4-OI- and DMF do not later lysate AnnexinA1 in A*bca1* siRNA transfected cells.** Expression of **A,B** *Anxa1,* **C,D)** *hmox1,* **E,F)** *Il1b* by qPCR from BMDMs pretreated with vehicle, 125 μM 4-OI or 20 μM DMF for two hours prior to stimulation with LPS (100 ng/mL) for 6 or 24 hours. (n=6 from 3 independent experiments).**G)** Lysate ABCA1 and **H**) annexin A1 quantified from western blots of BMDMs were transfected with 50 nM control siRNA or ABCA1 siRNA for 24 hours. The cells were then pretreated with 200 μM 4-OI or 25 μM DMF for two hours prior to stimulation with LPS (100 ng/mL) for 24 hours (*n* = 6 from three independent experiments). Data are presented as mean ± S.E.M and a one-way ANOVA was performed. The data show the adjusted p value obtained from multiple comparisons, corrected for using the Tukey test. *p < 0.05, **p < 0.005, ***p < 0.0005, ****p < 0.0001
